# Ipilimumab augments antitumor activity of bispecific antibody-armed T cells

**DOI:** 10.1186/1479-5876-12-191

**Published:** 2014-07-09

**Authors:** Hiroshi Yano, Archana Thakur, Elyse N Tomaszewski, Minsig Choi, Abhinav Deol, Lawrence G Lum

**Affiliations:** 1 Departments of Oncology, Wayne State University and Barbara Ann Karmanos Cancer Institute, 740.1 Hudson Webber Cancer Research Center, 4100 John R., Detroit, MI 48201, USA; 2 Medicine, Wayne State University and Karmanos Cancer Institute, Detroit, MI 48201, USA; 3 Immunology and Microbiology, Wayne State University and Karmanos Cancer Institute, Detroit, MI 48201, USA

**Keywords:** Ipilimumab, Cytotoxic T-lymphocyte antigen-4 (CTLA-4), Bispecific antibody, Pancreatic cancer, Colorectal cancer, Burkitt’s lymphoma, Activated T cells, Immunotherapy

## Abstract

**Background:**

Ipilimumab is an antagonistic monoclonal antibody against cytotoxic T-lymphocyte antigen-4 (CTLA-4) that enhances antitumor immunity by inhibiting immunosuppressive activity of regulatory T cells (Treg). In this study, we investigated whether inhibiting Treg activity with ipilimumab during *ex vivo* T cell expansion could augment anti-CD3-driven T cell proliferation and enhance bispecific antibody (BiAb)-redirected antitumor cytotoxicity of activated T cells (ATC).

**Methods:**

PBMC from healthy individuals were stimulated with anti-CD3 monoclonal antibody with or without ipilimumab and expanded for 10-14 days. ATC were harvested and armed with anti-CD3 x anti-EGFR BiAb (EGFRBi) or anti-CD3 x anti-CD20 BiAb (CD20Bi) to test for redirected cytotoxicity against COLO356/FG pancreatic cancer cell line or Burkitt’s lymphoma cell line (Daudi).

**Results:**

In PBMC from healthy individuals, the addition of ipilimumab at the initiation of culture significantly enhanced T cell proliferation (p = 0.0029). ATC grown in the presence of ipilimumab showed significantly increased mean tumor-specific cytotoxicity at effector:target (E:T) ratio of 25:1 directed at COLO356/FG and Daudi by 37.71% (p < 0.0004) and 27.5% (p < 0.0004), respectively, and increased the secretion of chemokines (CCL2, CCL3, CCL4,CCL5, CXCL9, and granulocyte-macrophage colony stimulating factor(GM-CSF)) and cytokines (IFN-γ, IL-2R, IL-12, and IL-13), while reducing IL-10 secretion.

**Conclusions:**

Expansion of ATC in the presence of ipilimumab significantly improves not only the T cell proliferation but it also enhances cytokine secretion and the specific cytotoxicity of T cells armed with bispecific antibodies.

## Introduction

Efficacy of cancer immunotherapy using targeted T cells has been limited by tumor-induced immunosuppression or regulatory T cells (Tregs) that interfere with T cell effector functions resulting in failure to induce robust cellular and humoral antitumor responses. Since recent clinical trial data show that cytotoxic T-lymphocyte antigen-4 (CTLA-4) positive Tregs are associated with reduced T cell antitumor activities [1], we hypothesized that treatment with anti-CTLA-4 monoclonal antibody (mAb) during T cell expansion would augment the proliferative and functional activities of activated T cells.

CTLA-4 is constitutively expressed on CD4^+^CD25^high^forkhead box P3^+^ (FOXP3^+^) Treg cells [2], transiently on newly activated T cells and minimally or negative on naïve T cells [3,4]. CTLA-4 is homologous to the co-stimulatory receptor CD28 but competes for CD80 and CD86 binding with much higher avidity [5-7] leading to suppression of T cell activation [8,9]. CLTA-4 blockade studies have demonstrated greater tumor regression in antibody-treated mice [10-12]. Furthermore, Treg-depletion prior to blocking the CTLA-4 signaling pathway results in enhanced T cell proliferation and tumor-specific cytotoxicity *in vivo*[2,13] and *in vitro*[2].

Ipilimumab (Yervoy®, Bristol-Myers Squibb) is a human mAb antibody directed at CTLA-4 that inhibits the binding of CLTA-4 to CD80 and CD86 during T cell activation, therefore increasing the CD28 binding opportunity to the co-stimulatory factors for appropriate T cell activation. In the FDA registration trial, ipilimumab significantly improved the median overall survival from 6.4 months in the group that received gp100 alone to 10.0 or 10.1 months in the group that received ipilimumab with or without gp100 (cancer vaccine comprises HLA-A*0201–restricted peptides derived from the melanosomal protein, glycoprotein 100 [gp100] [14]), respectively, in patients with unresectable stage III and IV melanoma [14]. However, 88.9% of the patients experienced dermatologic, gastrointestinal, or other immune-related adverse events (irAEs) from ipilimumab of which 10-15% were grade 3 or 4 that resulted in 2.1% drug-related deaths [14]. Therefore, new approaches that provide the antitumor effects of ipilimumab without the side effects are needed. One approach to avoid irAEs would be by adding ipilimumab at the initiation of T cell expansion cultures to enhance the proliferation and cytotoxicity mediated by BiAb-armed ATC. This study shows that ipilimumab enhances T cell proliferation, tumor-specific cytotoxicity mediated by BiAb, and cytokine secretion without Treg-depletion prior to ATC expansion. These results could be used to optimize antitumor activity in immunotherapeutic approaches for patients with oncological or hematological malignancies.

## Materials and methods

### Cell lines

The human Burkitt’s lymphoma cell line (Daudi) and human pancreatic cancer cell line (COLO356/FG) were maintained in RPMI-1640 or DMEM culture media (Lonza Inc., Allendale, NJ), respectively, supplemented with 10% fetal bovine serum (FBS) (Valley Biomedical Inc., Winchester, VA), 2 mM L-glutamine (Lonza Inc.), 50 IU/mL penicillin and 50 μg/mL streptomycin (Lonza Inc.).

### Generation of ATC

PBMC for ATC expansion were obtained from healthy volunteers (KCI protocol 2007-012) and leukapheresis samples from GI patients which were cryopreserved prior to immunotherapy (KCI protocol 2011-025). Both protocols were approved by the Wayne State University Human Investigation Committee. T cells in the PBMC were activated by 20 ng/mL of anti-CD3 monoclonal antibody (OKT3, Ortho Biotech, Horsham, PA) and expanded with 100 IU/mL of Interleukin-2 (IL-2) in RPMI-1640 supplemented with 10% FBS, 2 mM L-glutamine, 50 units/mL penicillin and 50 μg/mL streptomycin (complete RPMI-1640) for 14 days at 37°C with 5% CO_2_. At the initiation of culture, ipilimumab (YERVOY® by Bristol-Myer Squibb, Princeton, NJ) was added only once to the cultures at various concentrations (0, 0.5, 5.0, and 50 μg/mL). Cultures were fed every 2 to 3 days and maintained at a concentration of 1x10^6^ cells/mL. IL-2 was added at every feeding at 100 IU/mL.

### Production of bispecific antibodies and arming of ATC

Bispecific antibodies (BiAbs) were produced by chemical heteroconjugation of OKT3 and Rituxan (a humanized anti-CD20 IgG1, Genentech Inc., South San Francisco, CA) or Erbitux (a humanized anti-epidermal growth factor receptor (EGFR) IgG1, ImClone LLC., Branchburg, NJ) as described [15,16]. ATC were armed with anti-OKT3 x anti-CD20 BiAb (CD20Bi) or anti-OKT3 x anti-EGFR BiAb (EGFRBi) using a previously optimized concentration (50 ng/10^6^ ATC) [17].

### Cytotoxicity/^51^Cr release assay

To target adherent cells, COLO356/FG cells were plated in 96-well flat-bottom microtiter plates at 4x10^4^ cells/well and allowed to adhere overnight. The cells were labeled with ^51^Cr at 20 μCi/mL in the labeling media (50% FBS in complete RPMI-1640) for 5 hours at 37°C, and washed with complete RPMI-1640 to remove unincorporated isotope. For non-adherent cell targeting, Daudi cells were labeled with ^51^Cr at 100 μCi/10^6^ cells in a 15 mL conical tube for 4 hours at 37°C, washed with complete RPMI-1640, and plated in 96-well round-bottom microtiter plates at 1x10^4^ cells/well. Effectors (unarmed ATC and aATC) were then added to achieve effector:target (E:T) ratios of 25:1 and 12.5:1. Co-cultures were incubated for 4 hours (Daudi) or 18 hours (COLO356/FG) and the supernatant was collected for liquid scintillation counting to quantitate the amount of released ^51^Cr. Percent cytotoxicity was calculated as follows: (experimental cpm – spontaneous cpm)/(maximum cpm – spontaneous cpm) × 100 [15]. Means and standard errors were calculated from four to six replicates per sample.

### T cell sub-population profiling

Changes in T cell sub-populations were quantitated by flow cytometric analysis as described [18,19]. CD8-PE-Cy5, CD16-PE, CD25-APC, CD45RA-FITC, CD45RO-PE, CD56-APC, CD127-PE, and CD152-PE-Cy5 were purchased from BD Biosciences (San Jose, CA). CD3-FITC and CD4-APC were purchased from Miltenyi Biotec (Auburn, CA).

### Quantitation of released cytokines in co-cultures

ATC expanded with or without ipilimumab were armed with EGFRBi and co-cultured with COLO356/FG at 10:1 E:T ratio. The amount of cytokines released by unarmed ATC or aATC in the co-culture supernatant was quantitated using 25-plex human cytokine Luminex Assay (Invitrogen, Carlsbad, CA) in the Bio-Plex System (Bio-Rad Lab., Hercules, CA) as described [19,20]. Analysis panel includes IL-1β, IL-1 receptor antagonist (IL-1Ra), IL-2, IL-2R, IL-4, IL-5, IL-6, IL-7, IL-8, IL-13, IL-17, tumor necrosis factor (TNF)-α, interferon (IFN)-α, IFN-γ, granulocyte macrophage colony-stimulating factor (GM-CSF), macrophage inhibitory protein (MIP)-1α (CCL3), MIP-1β (CCL4), interferon inducible protein (IP)-10, monokine induced by IFN-γ (MIG or CXCL9), eotaxin, regulated on activation normal T cell expressed and secreted (RANTES or CCL5), and monocyte chemotactic protein (MCP)-1 (CCL2). Bio-Plex Manager Software was used to calculate the cytokine concentrations using a recombinant cytokines-derived standard curve.

### Statistical analysis

Quantitative data are presented as the means of at least three or more independent experiments with standard errors. Paired, two-tailed *t*-test and matched sample one-way ANOVA were used to determine whether the data were statistically significant.

## Results

### Ipilimumab affects T cell effector functions in dose dependent manner

**Figure 1:**
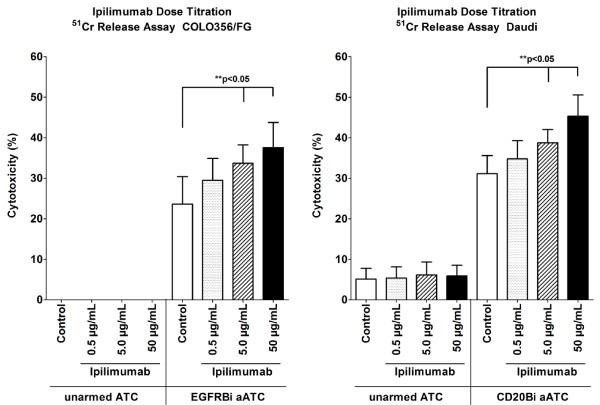
**Dose dependent enhancement of anti-tumor cytotoxicity of expanded ATC by ipilimumab.** Effect of ipilimumab on BiAb-mediated T cell cytotoxicity was examined by adding ipilimumab at the initiation of ATC expansion cultures from healthy donors at various concentrations ranging from 0 to 50 μg/mL. ATC were harvested on day 14, armed with EGFRBi or CD20Bi, and co-cultured at 25:1 E:T ratio for 18 hours with COLO356/FG **(*left***) or 4 hours with Daudi **(*right*)** for cytotoxicity assay. BiAb-mediated tumor specific cytotoxicity was measured by ^51^Cr release assay. Each bar represents a mean ± SE of 3 donors (COLO356/FG) or 4 donors (Daudi). The dose-dependent effect of ipilimumab was statistically significant in matched sample, one-way ANOVA for COLO356/FG (*p < 0.05) and Daudi (**p < 0.05).

In order to determine the optimal *in vitro* concentration of ipilimumab, concentrations equivalent to *in vivo* dosage (3mg/kg) [14] for an average body weight (~75-88kg) [21] were tested in the ATC expansion culture by dose titration. T cells in PBMC derived from three healthy donors were activated by anti-CD3 mAb and expanded with IL-2 for 10-14 days. The expansion cultures were initiated with 0 (control), 0.5, 5.0, and 50 μg/mL of ipilimumab. ATC were harvested and armed with CD20Bi or EGFRBi to target CD20 positive Burkitt’s lymphoma cell line (Daudi) or EGFR positive pancreatic cancer cell line (COLO356/FG), respectively. Cytotoxicity was measured by ^51^Cr release assay at effector:target (E:T) of 25:1. In both COLO356/FG and Daudi targeting, tumor-specific cytotoxicity was significantly enhanced (p < 0.05) in a dose dependent manner (Figure 1). The highest T cell cytotoxicity was observed at 50 μg/mL of ipilimumab with mean percent increases of 69.8% and 49.0% for EGFRBi and CD20Bi targeting, respectively. Based on these results, a dose of 50 μg/mL was chosen for all the subsequent experiments unless otherwise indicated.

### Enhanced cytotoxicity is sustained at lower E:T

**Figure 2:**
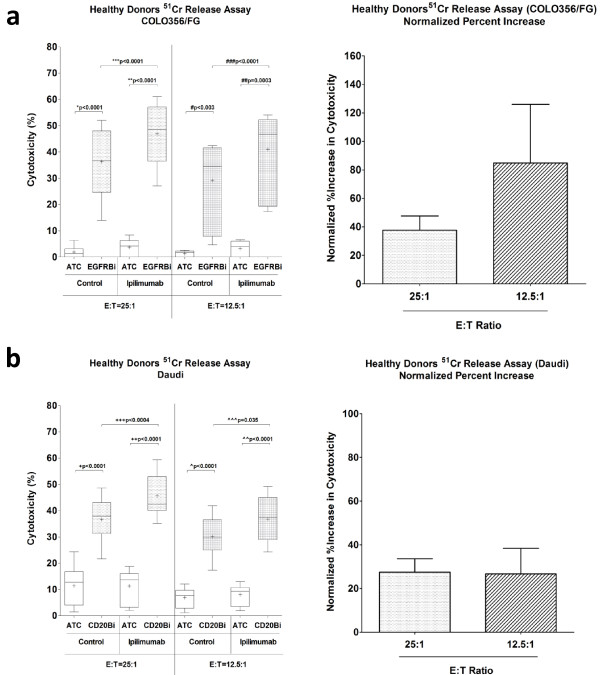
**Enhanced BiAb mediated cytotoxicity in ipilimumab supplemented ATC of healthy donors. (a)** T cells in PBMC derived from healthy donors were expanded with 0 (control) or 50 μg/mL of ipilimumab, and ATC were harvested, armed and tested for BiAb-mediated tumor specific cytotoxicity. EGFRBi armed ATC and COLO356/FG were co-cultured for 18 hours at E:T ratios of 25:1 (n = 9) and 12.5:1 (n = 7), and difference in the specific cytotoxicity was measured by ^51^Cr release assay **(*left*)**. Cytotoxicity of EGFRBi armed ATC in the ipilimumab group was normalized to the control group to calculate the percent increase **(*right*)**. **(b)** CD20Bi armed ATC and Daudi were co-cultured for 4 hours at E:T ratios of 25:1 (n = 11) and 12.5 (n = 8), and specific cytotoxicity was measured by ^51^Cr release assay **(*left*)**. Cytotoxicity of CD20Bi armed ATC in the ipilimumab group was normalized to the control group to calculate the percent increase **(*right*)**. Each bar represents a mean ± SE for ≥ 7 samples. The bar within each box shows a median while + symbol represents a mean for each data set. For each set of data, a paired two-tailed *t* test was performed.

In order to confirm the ability of ipilimumab to enhance the specific cytotoxicity at lower E:T, ^51^Cr release assay was repeated with healthy donors using the 50 μg/mL dose of ipilimumab. Consistent with the dose titration results, ipilimumab significantly enhanced the BiAb-mediated tumor specific cytotoxicity at E:T of 25:1 and 12.5:1 with both EGFRBi and CD20Bi, targeting COLO356/FG and Daudi, respectively (Figure 2a and b). The mean percent increases in the cytotoxicity were 37.7% (p = 0.0001) and 27.5% (p < 0.0004) against COLO356/FG and Daudi. The enhanced cytotoxicity was sustained at the lower E:T of 12.5:1, and EGFRBi aATC had an even higher percent increase of 84.9% (p < 0.0001) (Figure 2a).

### Increased cytotoxicity by BiAb-armed T cells from patients with gastrointestinal (GI) cancers in the presence of ipilimumab

**Figure 3:**
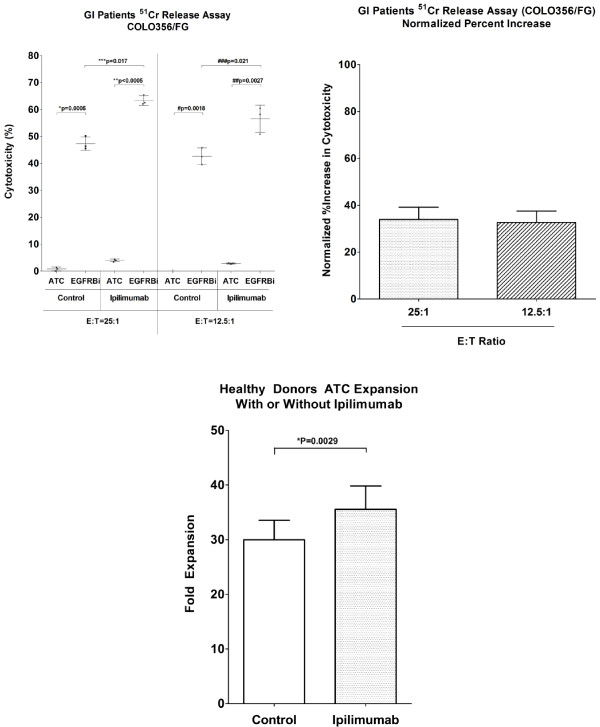
**Ipilimumab enhanced BiAb-mediated cytotoxicity of ATC derived from patients with gastrointestinal cancer.** T cells in cryopreserved PBMC of 3 patients with gastrointestinal cancer were expanded with 0 (control) or 50 μg/mL ipilimumab, and ATC were harvested on day 14, armed with EGFRBi, and tested for the tumor specific cytotoxicity (n = 3). **(*left*)**: EGFRBi armed ATC and COLO356/FG were co-cultured at E:T ratios of 25:1 and 12.5:1 E:T ratios. For each group, a paired two-tailed *t* test was performed. **(*right*)**: Cytotoxicity of EGFRBi armed ATC in the ipilimumab group was normalized to the control group to calculate the percent increase. Each bar represents a mean ± SE is shown for 3 patients. For each set of data, paired, two-tailed *t* test was performed. **(*lower*)**: T cells in PBMC derived from healthy donors were activated with anti-CD3 stimulation and expanded with IL-2 in the presence of 0 (control) or 50 μg/mL ipilimumab (n = 18). Cell counts were performed at each feeding to determine the T cell proliferation. The bars represent means for each data set (paired two-tailed *t* test).

Since T cells obtained from cancer patients may be defective in antitumor function due to an immunosuppressive tumor microenvironment *in vivo*[22-27], we tested whether ipilimumab could enhance cytotoxicity in T cells from GI patients. T cells from cryopreserved leukapheresis products of three patients with gastrointestinal (2 colorectal and 1 pancreatic) cancers were activated by anti-CD3 mAb with or without ipilimumab and expanded for 14 days with IL-2. Harvested ATC were armed with EGFRBi and tested for cytotoxicity against COLO356/FG at E:T of 25:1 and 12.5:1. Ipilimumab clearly enhanced the EGFRBi-mediated tumor specific cytotoxicity by ATC derived from GI patients (Figure 3, left panel). The mean percent increases were 33.9% (p = 0.017) and 32.7% (p = 0.021) at E:T of 25:1 and 12.5:1, respectively (Figure 3, right panel).

### Ipilimumab enhances proliferation of activated T cells

Both *in vivo* and *in vitro* studies have shown that blocking CTLA-4 signaling enhances T cell proliferation, and in some cases, synergistic enhancement was observed when Treg cells were depleted prior to T cell stimulation [2,13]. We examined an effect of ipilimumab on the proliferation of ATC from PBMC without physical Treg-depletion. T cell proliferation was monitored by measuring the cell concentrations each time the cultures were fed. As shown in the lower panel of Figure 3, ATC proliferation increased by 18.61% (p = 0.0029) in the presence of ipilimumab.

### Ipilimumab decreases CD4/CD8 ratio and Treg population in expanded ATC

**Figure 4:**
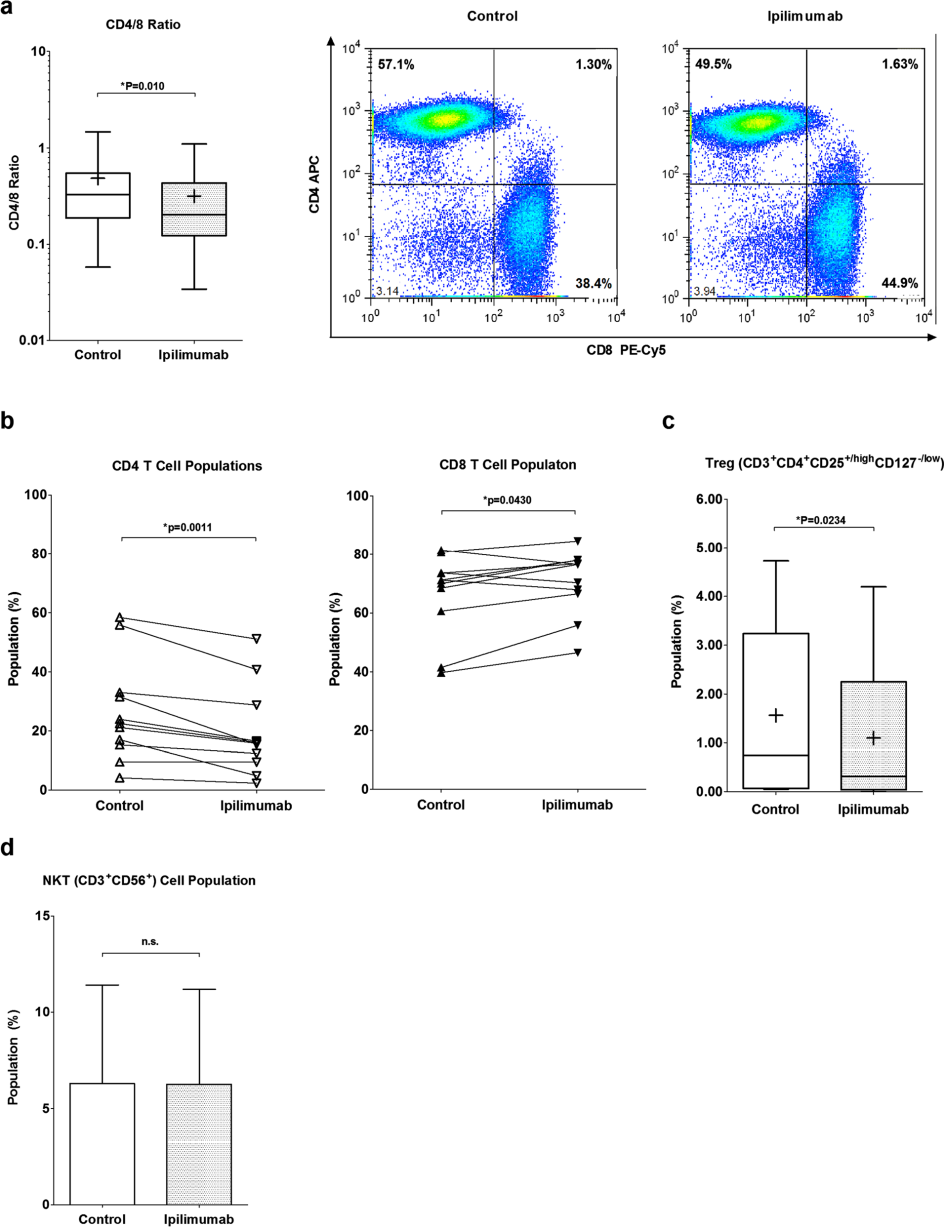
**Ipilimumab altered the T cell-subpopulation profile in expanded ATC. (*a*)**: ATC expanded with ipilimumab had a decreased CD4/8 ratio (n = 11) **(*left*)**. The panel summarizes 11 experiments (n = 11); the bars in the boxes are medians while + symbols represent means. One representative flow cytometry analysis is shown **(*right*)**. **(*b*)**: The proportion of CD4 and CD8 T cells were determined in the presence or absence of ipilimumab. Control and ipilimumab groups in each of 11 experiments are connected with a trend line. **(*c*)**: The expanded 8 ATC products were analyzed by flow cytometry to show the change in the Treg population (CD3^+^CD4^+^CD25^high/+^CD127^low/-^). The bars in the boxes are medians while + symbols represent means. **(*d*)**: NKT cell population (CD3^+^CD56^+^) in the expanded ATC products is shown. Each bar represents a mean with standard error of eight experiments (n = 8). Each bar represents a mean ± SE of 8 experiments, and paired, two-tailed *t* test was performed.

Recent clinical studies demonstrated that patients who receive ipilimumab exhibit increased proliferation of both CD4 and CD8 T cells [28,29]. Based on the enhanced cytotoxicity and the increased proliferation of ATC in our study, we hypothesized that ipilimumab may enhance T cell cytotoxicity by preferentially promoting CD8 T cell expansion. Expanded ATC were harvested after 14 days of culture, and the changes in T cell subpopulations were quantitated by flow cytometry. The CD4/CD8 ratio was significantly decreased (p = 0.010) in the presence of ipilimumab, indicating an increase in CD8 population (Figure 4a). To clarify the changes responsible for the decreased CD4/CD8 ratio, CD4 and CD8 T cell populations were separately quantitated (Figure 4b). Proportion of CD4^+^ cells decreased (p = 0.0011) while proportion of CD8^+^ cells increased (p = 0.0430). There was no significant difference in CD4CD8 double positive T cells (data not shown). Furthermore, when the proportion of Treg cells between control and ipilimumab-supplemented ATC preparations were compared, there was a statistically significant reduction in the Treg population (n = 8, p = 0.0234) in the ipilimumab group (Figure 4c).

### Secretion of immune activating chemokines and cytokines were elevated by ipilimumab

**Figure 5:**
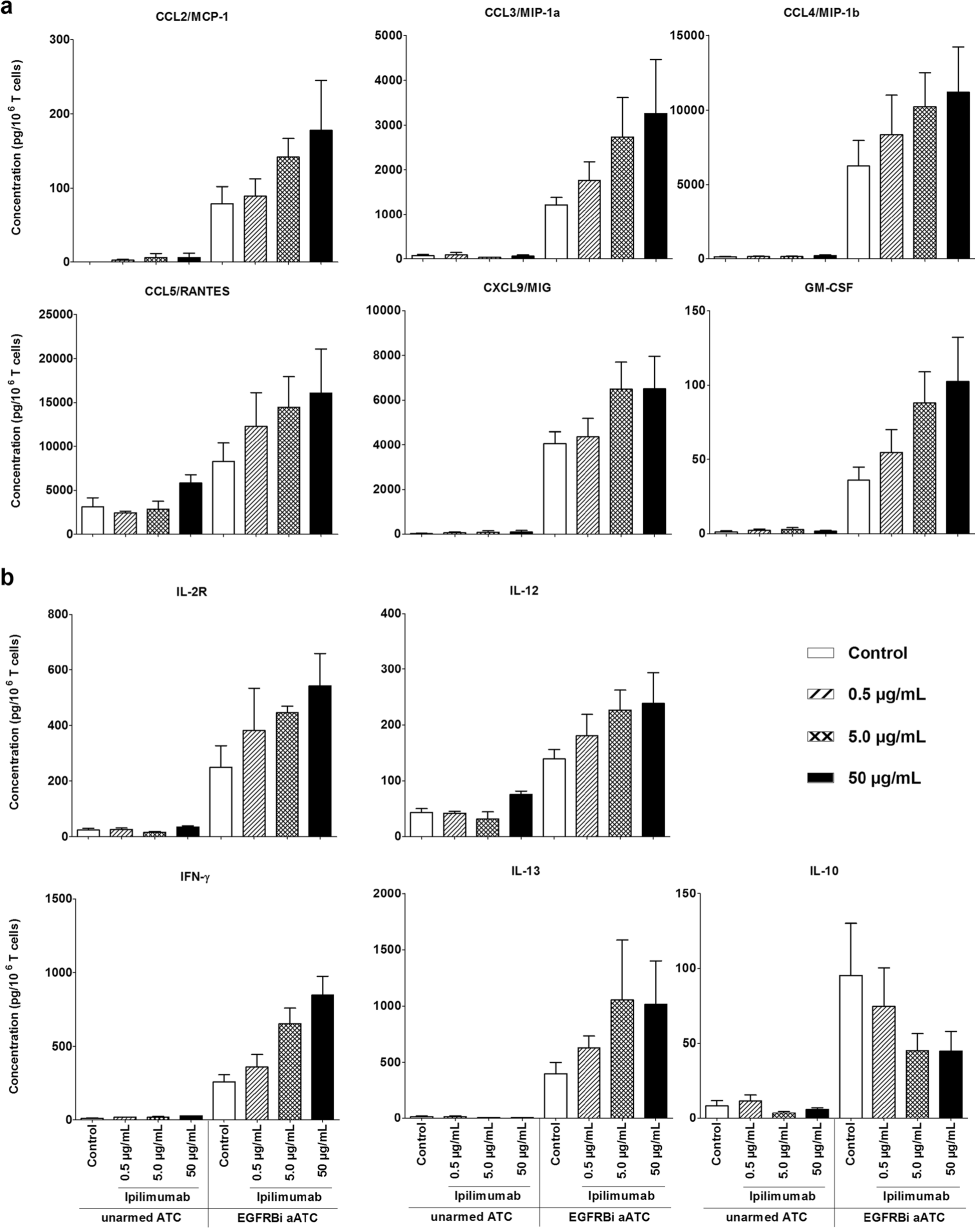
**The pattern of dose-dependent changes in cytokine secretion levels in ATC expanded with Ipilimumab.** Anti-CD3 activated T cells in PBMC derived from 3 healthy donors were cultured in the presence of 0 (control), 0.5, 5.0 and 50 μg/mL ipilimumab for 14 days. Harvested ATC were armed with EGFRBi and co-cultured with COLO356/FG target cells at 10:1 E:T for 24 hours. The supernatants were collected and quantitated in the multi-plex Luminex assay. **(*a*)**: Shows changes in the secretion levels of CCL2, CCL3, CCL4, CCL5, CXCL9, and GM-CSF. **(*b*)**: Shows changes in the secretion levels of IL-2R, IFN-γ, IL-12, and IL-13. Each bar represents a mean ± SE of 3 healthy donors (n = 3).

ATC were expanded with ipilimumab at 0, 0.5, 5.0 and 50 μg/mL concentrations to determine whether ipilimumab could alter the cytokine secretion levels in a dose dependent manner. The harvested ATC were armed with EGFRBi and co-cultured with COLO356/FG at 10:1 E:T for 24 hours and supernatants were harvested. Among 25 different cytokines and chemokines in the analysis panel, there was a pattern of dose dependent increase in the secretion of chemokines CCL2, CCL3, CCL4, CCL5, CXCL9, and GF-CSF (Figure 5a). Cytokine analysis of the cultures also revealed that ATC expanded in the presence of ipilimumab secreted increased amount of IFN-γ, IL-2R, IL-12, and IL-13, whereas the secretion of IL-10 was decreased (Figure 5b).

## Discussion

Previously we have shown that T cells expanded from the leukapheresis product or PBMC from the immunosuppressive environment of cancer patients can be stimulated to improve their functions *in vitro* as well as *in vivo*[30,31]. ATC armed with anti-CD3 x anti-tumor associated antigen BiAbs exhibit high levels of specific cytotoxicity against tumor cells expressing Her2/neu [17,18,32], CD20 [15], GD2 [33], and EGFR [16,19] via redirected non-MHC-restricted perforin/granzyme-dependent killing. Furthermore, arming with BiAbs creates artificial cytotoxic T lymphocytes wherein TCR engagement via BiAb-bridge between the armed ATC and the antigen on the tumor cells induces release of significantly higher levels of IFN-γ, IL-2R, IL-12, CCL3, CCL4 and CXCL9, compared to unarmed ATC used as controls [19]. In this study, we demonstrated that the addition of ipilimumab at the initiation of culture significantly improves T cell proliferation, BiAb-mediated tumor-specific cytotoxicity, and synthesis of inflammatory chemokines and cytokines. Phenotypic analysis of T cell subpopulations showed a decreased Tregs population with a concomitant decrease in IL-10 secretion as well as a reduced CD4/CD8 ratio in the ATC products expanded in the presence of ipilimumab. The reduced CD4/CD8 ratio may account for the increased specific cytotoxicity. These data demonstrate that ipilimumab can improve ATC proliferation and effector functions while it diminishes immune suppressive Treg activity.

Enhanced T cell proliferation in the presence of ipilimumab may be due to the inhibition of the interaction between pre-existing Treg cells and monocytes/dendritic cells in PBMC that were indirectly activated upon anti-CD3 stimulation of T cells. In addition, it is likely that ipilimumab blocked the CTLA-4-mediated inhibitory signaling in activated effector T cells as CTLA-4 expression is known to peak around 2 days after anti-CD3 stimulation [3,4]. However, it is not clear whether decreased CD4 percent population was due to a direct effect of ipilimumab on CD4 T cell proliferation or due to an enhanced CD8 T cell expansion induced by ipilimumab.

As shown in the Figure 4d, the NKT cell population in some healthy donors persisted during the ATC culture period in both control and ipilimumab ATC preparations with no significant difference. NK cell activation is a complex set of signal transduction pathways including direct cell-cell signaling from APCs and humoral signaling via cytokines and chemokines [34]. Since the ATC culture was fed with IL-2, which is a NK cell stimulatory factor [34,35], and antigen-bound IgG is also known to activate NK and NKT cells via Fcγ receptor bindings [34], NKT cells may be activated by IL-2 and Fc binding of ipilimumab bound to CTLA-4 on the surface of ATC which in turn may have exhibited the elevated secretion of INF-γ and CTL activity. Additional studies with purified NKT cells will be needed to address the effects of ipilimumab on NK and NKT cells.

The cytokine quantitation studies revealed that ipilimumab enhances the secretion of various cytokines and chemokines in a dose-dependent manner. Dose dependent (ipilimumab) increase in IFN-γ and IL-12 suggest generation of Th1 cytokines produced during armed ATC mediated killing of target cells. These cytokines and chemokines may activate endogenous immune cells *in vivo*[30,31]. However, the role of soluble IL-2R (sIL-2R) in culture supernatant is not clear. Both Tregs and activated T cells can release IL-2R, and measurable levels of sIL-2R in the blood have been shown to indicate sustained immune activation [36]. On the other hand, sIL-2R also competes for IL-2 binding on activated T cells, thereby inhibiting T cell proliferation [36,37]. Increased release of sIL-2R has also been shown to induce differentiation of CD4 T cells into FOXP3^+^ Treg [38]. Since proliferation and activation were both enhanced in the presence of ipilimumab, increased levels of sIL-2R in this study are likely due to the activation of T cells rather than its immune suppressive activity.

Intriguingly, decreased levels of IL-10 suggest that ipilimumab attenuates the release of IL-10 in dose-dependent manner. IL-10 has been shown to contribute in tumor derived immune suppression [39] by suppressing CD4 T cells [40] and inducing myeloid derived suppressor cells [41].

**Figure 6:**
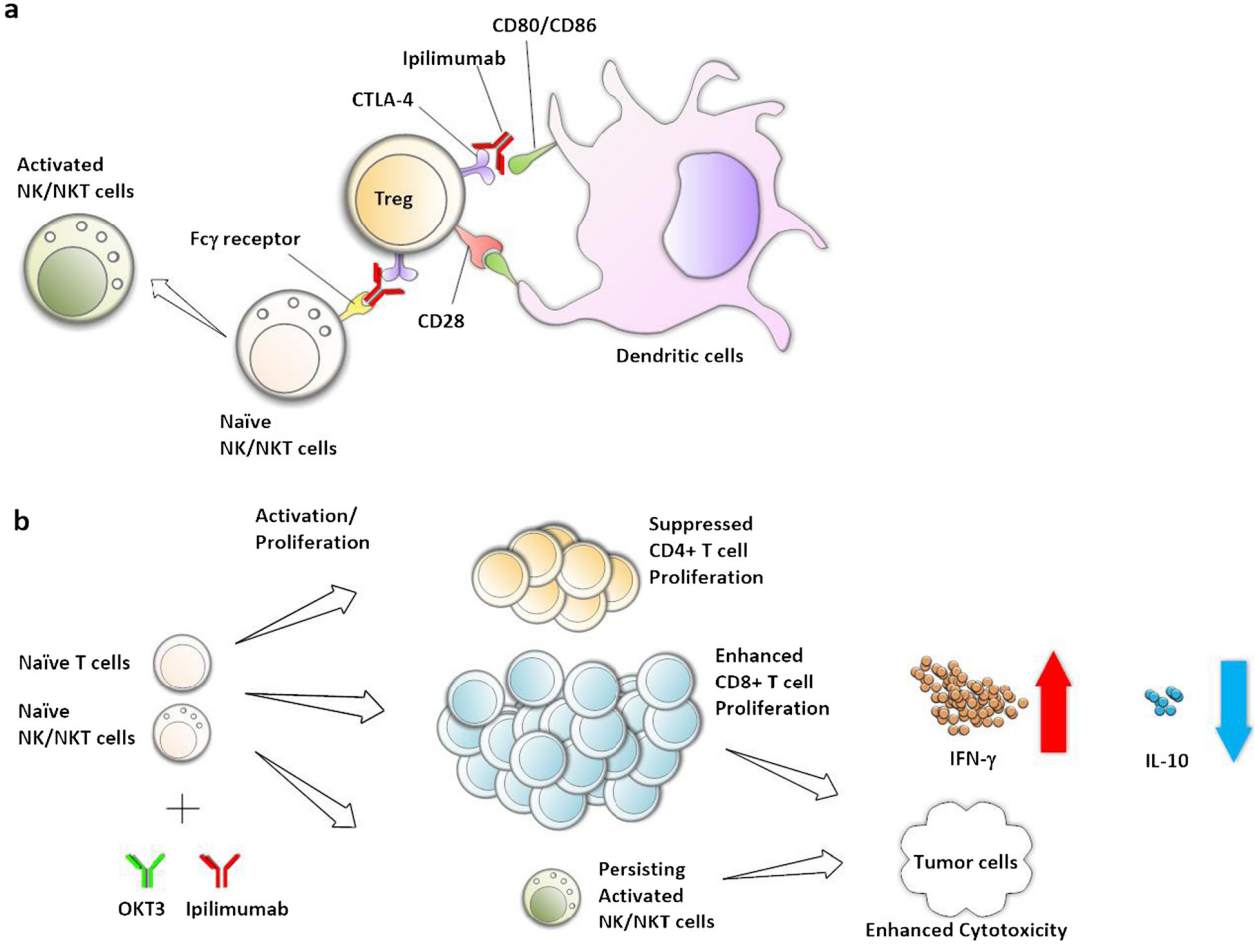
**A schematic summary of our study results showing a pivotal role that ipilimumab may play in modulating T cell and NK/NKT cell activation. (a)** CTLA-4 on the surface of Treg or early activated T cells competes with CD28 in binding with co-stimulatory factors CD80 or CD86 on antigen presenting cells. **(b)** Ipilimumab enhances proliferation of CD8^+^ T cells while proliferation of CD4^+^ T cells was reduced, hence the decreased CD4/CD8 ratio. The enhanced CD8^+^ T cell proliferation resulted in the improved BiAb-mediated antitumor cytotoxicity in both Daudi and COLO356/FG models. The enhanced antitumor activity was consistently observed in elevated secretion of IFN-γ and decreased IL-10 secretion. Although there was no difference between control and ipilimumab groups in NKT cell proliferation, ipilimumab-mediated NKT cell activation may have contributed in the increased cytotoxicity and IFN-γ secretion.

The increased chemokine secretion suggests that ipilimumab can promote not only the cytotoxicity of the expanded ATC but also their ability to recruit endogenous immune cells [30,31] such as memory T cells, monocytes, dendritic cells, macrophages, NK cells, and neutrophils. These data further indicates that ipilimumab can augment effector activities of ATC while inhibiting the regulatory components of the immune system (Figure 6).

In summary, we demonstrated that ipilimumab can improve ATC proliferation, enhance the BiAb-mediated tumor-specific cytotoxicity, and increase cytokine synthesis, while it attenuates Treg activity as shown by decreased level of IL-10 secretion and reduced Treg population. This mechanism may be clinically relevant since ipilimumab treatment of PBMC from GI patients was able to enhance BiAb-mediated cytotoxicity of ATC directed at pancreatic cancer cell line COLO356/FG. The approach of using ipilimumab to optimize effector functions of *ex vivo* expanded ATC may improve the efficacy of BiAb-mediated antigen targeted adoptive T cell immunotherapy without increasing ipilimumab related toxicities.

## Abbreviations

APC: Antigen presenting cells; ATC: Activated T cells; BiAb: Bispecific antibody; CTLA-4: Cytotoxic T-lymphocyte antigen-4; GI cancer: Gastrointestinal cancer; CCL: Chemokine (C-C motif) ligand; CXCL: Chemokine (C-X-C motif) ligand; DMEM: Dulbecco’s modified Eagle’s medium; EGFR: Epidermal growth factor receptor; FBS: Fetal bovine serum; FDA: The Food and Drug Administration; GD2: Disialoganglioside; GM-CSF: Granulocyte-macrophage colony-stimulating factor; IFN: Interferon; IL: Interleukin; MCP: Monocyte chemotactic protein; MIP: Macrophage inhibitory protein; TNF: Tumor necrosis; IP: Interferon inducible protein; MIG: Monokine induced by IFN-γ; RANTES: Regulated on activation, normal T cell expressed and secreted; NK: Natural killer cells; NKT: Natural killer T cells; Th: Helper T cells; PBMC: Peripheral blood mononuclear cells; RPMI: Roswell Park Memorial Institute.

## Competing interests

LGL is a co-founder of Transtarget Inc. AD receives research funding from BMS. HY, AT, and MC have no competing interests.

## Authors’ contributions

HY designed the study, performed experiments, analyzed data and statistics, and wrote the manuscript. AT participated in the cytokine assay and data analysis. AT, LGL MC and AD participated in the design of the study and in drafting the manuscript. All authors read and approved the final manuscript.
